# Peptidylarginine Deiminase Inhibitor Application, Using Cl-Amidine, PAD2, PAD3 and PAD4 Isozyme-Specific Inhibitors in Pancreatic Cancer Cells, Reveals Roles for PAD2 and PAD3 in Cancer Invasion and Modulation of Extracellular Vesicle Signatures

**DOI:** 10.3390/ijms22031396

**Published:** 2021-01-30

**Authors:** Pinar Uysal-Onganer, Stefania D’Alessio, Maria Mortoglou, Igor Kraev, Sigrun Lange

**Affiliations:** 1 Cancer Research Group, School of Life Sciences, University of Westminster, London W1W 6 UW, UK; w1754188@my.westminster.ac.uk; 2 Tissue Architecture and Regeneration Research Group, School of Life Sciences, University of Westminster, London W1W 6 UW, UK; w1650366@my.westminster.ac.uk; 3 Electron Microscopy Suite, Faculty of Science, Technology, Engineering and Mathematics, Open University, Milton Keynes MK7 6AA, UK; igor.kraev@open.ac.uk

**Keywords:** peptidylarginine deiminases (PADs), protein deimination, extracellular vesicles (EVs), pancreatic ductal adenocarcinoma (PDAC), moesin, prohibitin (PHB), deiminated histone H3 (citH3), microRNA (miR-21; miR-221; miR-126)

## Abstract

Pancreatic ductal adenocarcinoma (PDAC) is one of the most aggressive malignancies with limited survival rate. Roles for peptidylarginine deiminases (PADs) have been studied in relation to a range of cancers with roles in epigenetic regulation (including histone modification and microRNA regulation), cancer invasion, and extracellular vesicle (EV) release. Hitherto though, knowledge on PADs in PDAC is limited. In the current study, two PDAC cell lines (Panc-1 and MiaPaCa-2) were treated with pan-PAD inhibitor Cl-amidine as well as PAD2, PAD3, and PAD4 isozyme-specific inhibitors. Effects were assessed on changes in EV signatures, including EV microRNA cargo (miR-21, miR-126, and miR-221), on changes in cellular protein expression relevant for pancreatic cancer progression and invasion (moesin), for mitochondrial housekeeping (prohibitin, PHB), and gene regulation (deiminated histone H3, citH3). The two pancreatic cancer cell lines were found to predominantly express PAD2 and PAD3, which were furthermore expressed at higher levels in Panc-1, compared with MiaPaCa-2 cells. PAD2 isozyme-specific inhibitor had the strongest effects on reducing Panc-1 cell invasion capability, which was accompanied by an increase in moesin expression, which in pancreatic cancer is found to be reduced and associated with pancreatic cancer aggressiveness. Some reduction, but not significant, was also found on PHB levels while effects on histone H3 deimination were variable. EV signatures were modulated in response to PAD inhibitor treatment, with the strongest effects observed for PAD2 inhibitor, followed by PAD3 inhibitor, showing significant reduction in pro-oncogenic EV microRNA cargo (miR-21, miR-221) and increase in anti-oncogenic microRNA cargo (miR-126). While PAD2 inhibitor, followed by PAD3 inhibitor, had most effects on reducing cancer cell invasion, elevating moesin expression, and modulating EV signatures, PAD4 inhibitor had negligible effects and pan-PAD inhibitor Cl-amidine was also less effective. Compared with MiaPaCa-2 cells, stronger modulatory effects for the PAD inhibitors were observed in Panc-1 cells, which importantly also showed strong response to PAD3 inhibitor, correlating with previous observations that Panc-1 cells display neuronal/stem-like properties. Our findings report novel PAD isozyme regulatory roles in PDAC, highlighting roles for PAD isozyme-specific treatment, depending on cancer type and cancer subtypes, including in PDAC.

## 1. Introduction

Pancreatic ductal adenocarcinoma (PDAC) is a major cause of cancer-associated deaths in Western countries and is the eighth main source of cancer-related deaths globally [1]. Despite advances in diagnostic technology, PDAC is usually diagnosed late and at an incurable stage, while early diagnosis is directly linked to improved survival [2]. Additionally, only a small portion of PDAC patients can benefit from chemotherapy and especially in advanced stages, the chemotherapeutics options are narrow with gemcitabine being the first drug treatment with improvement in the median survival only by a few weeks [3]. Moreover, while carbohydrate antigen 19-9 (CA 19-9) has been the most common diagnostic biomarker for PDAC in the last 30 years [4], it cannot be characterised as a specific biomarker, especially for asymptomatic patients [5]. Therefore, it is of great importance to identify novel molecular markers and pathways to aid biomarker discovery, pancreatic cancer diagnostics, and for development of novel treatment options.

Peptidylarginine deiminases (PADs) are a group of calcium-dependent enzymes that cause post-translational deimination/citrullination in target proteins, leading to changes in their structure and function, affecting protein–protein interactions, generation of neo-epitopes, and modulating gene regulation [6,7,8]. This post-translational modification may also aid protein moonlighting, a phylogenetically conserved mechanism that allows proteins to carry out numerous functions within one polypeptide chain, in relation to both physiological and pathophysiological functions [9,10]. PADs’ roles in numerous autoimmune and inflammatory conditions are well acknowledged, and in cancers PADs have been assessed for causing changes in epigenetic regulation, in relation to modulatory effects on EV communication as well as cancer invasion [11,12,13,14,15,16,17]. In mammals five isozyme-specific PADs are described [6], which differ in their preference for protein targets and show tissue-specific expression. Therefore, a difference in prominence of the three main PAD isozymes (PAD2, 3, and 4) related to cancers is a topic of current interest, in relation to isozyme-specific targeting for specific cancer types, including heterogenous cancers as well as cancer subtypes [15,17]. Indeed, prominent roles for specific PAD isozymes have been associated with various cancers, including differences in modulating cell invasion proteins, effects on epigenetic regulation, and changes in EV signatures [11,12,15,17]. Currently, knowledge on PADs in PDAC is very limited and warrants further investigation.

EVs are mediators of cell communication in physiological processes, but also in cancers, where they are key mediators for intra/inter-tumour communication by transferring proteins, enzymes, and nucleic acids (mRNA, miRNA, lncRNA, sncRNA) to surrounding cells as part of their cargo [17,18,19,20]. EVs are lipid bilayer-enclosed structures, with a diameter of 30–1000 nm, and as they are released from their cells of origin they can be valid prognostic and diagnostic biomarkers. Due to EVs’ important roles in cancers, they are widely studied in a range of cancers, including also pancreatic cancer [21,22]. EVs released from cancer cells participate in the tumour’s intercellular communication, can influence the tumour microenvironment to promote angiogenesis, tumour growth, invasion and metastasis, as well as contribute to tumour metabolism [23,24,25]. Therefore, strategies to modulate EV signatures in cancer cells have been a focus of numerous studies, both with regard to limiting tumour growth in vivo, as well as sensitising cancer cells to chemotherapy [12,13,15,26,27,28,29,30,31,32,33]. The effect of PADs on modulating EV signatures in cancer cells has been highlighted in a number of studies, assessing both pan-PAD inhibitor Cl-amidine as well as PAD2, PAD3, and PAD4 isozyme-specific inhibitors [11,12,15,17]. As this has shown effects on sensitising cancer cells to chemotherapy, alongside effects on pro- and anti-oncogenic microRNA EV cargo, novel interventions for targeting EV communication in pancreatic cancer may also be of considerable interest.

microRNAs (miRs) are small (18 to 24 nucleotides long), endogenous, non-coding, evolutionary conserved, single-stranded RNA molecules, which can moderate gene expression at the post-transcriptional level through the binding to the complementary sequences of their target mRNAs at the 3′ untranslated regions (UTRs) [34]. More recent evidence has shown that the aberrant expression of miRs plays a significant role in numerous human malignancies and is also associated with poor prognosis, invasion, metastasis, and chemoresistance of PDAC [35]. Several signalling pathways associated with specific miRs are indeed related to PDAC progression. Specifically, upregulation of miR-21 and downregulation of miR-126 have been considered to contribute to PDAC progression, through the post-transcriptional upregulation of KRAS [36]. It has been suggested that activated KRAS (G12D) stimulates the miR-21 promoter in human PDAC cells [37] and that miR-21 is involved in oncogenic RAS-induced cell proliferation [38]. Moreover, miR-21 indirectly targets KRAS, while downregulation of miR-126 directly regulates KRAS signalling pathway and controls KRAS protein translation [39]. Aberrant expression of miR-126 in PDAC is associated with HER2 overexpression [40,41], which is related to more than 30% of PDAC cases [42]. While the HER2/neu signalling pathway was found to be involved in PDAC progression, specific inhibition of Her2/neu pathway did, however, not affect the overall survival [43]. In breast cancer, upregulation in miR-21 is also linked to positive HER2 status, larger tumour size, higher tumour stage and grade, and poor patient survival [44]. Furthermore, miR-21 is involved in the MAPK signalling pathway, which is important for the progression of pancreatic intraepithelial neoplasia (PanIN [45]). Overexpression of miR-21 has been shown to moderate cell proliferation and apoptosis of PDAC cells through the inhibition of both MAPK/ERK and PI3K/AKT signalling pathways [46]. Expression of miR-21 is also associated with the TGF-β signalling pathway, which is dysregulated in 80% of PDAC cases [47,48]. TGF-β is also associated with pathogenesis of PDAC at later stages and inactivation of tumour suppressor genes, including SMAD4, which is involved in several biological processes such as cell proliferation, differentiation, metastasis, and apoptosis [49,50]. miR-221 is one of the most dysregulated miRs in PDAC alongside miR-21 [51,52,53]. Upregulation of miR-221 has been found in a number of malignancies, including hepatocellular carcinoma, prostate adenocarcinoma, and colorectal carcinoma [54,55,56,57]. Moreover, a higher level of circulating miR-221 expression was detected in PDAC patients compared with patients with benign pancreatic tumours or healthy controls [58]. Upregulation of miR-221 also plays a significant role in platelet-derived growth factor (PDGF)-mediated epithelial to mesenchymal transition (EMT) phenotype, migration, metastasis, and uncontrolled proliferation of PDAC cells [59]. Additionally, miR-221 promotes the proliferation of PDAC cells through PTEN-Akt signalling pathway [53]. Overexpression of miR-221 can result in uncontrolled proliferation of PDAC cells through the inhibition of both MAPK and TGF-β signalling pathways [60].

We have previously shown that both pan-PAD inhibitor Cl-amidine and PAD2, 3, and 4 isozyme-specific inhibitors modulate a range of pro- and anti-oncogenic miRs in cancers such as glioblastoma multiforme (GBM), as well as in cancer cell-derived EVs, including miR-21 and miR-126 [15,17]. Therefore it may be of great importance to identify whether such PAD-mediated effects may also affect miR expression and EV-mediated miR export in other types of cancers, including in PDAC. In previous studies, PAD inhibitors were furthermore found to affect cancer cell invasion proteins including moesin, which is a critical factor for cell migration and filopodia formation [61]. Interestingly, in pancreatic cancer, moesin was found to be strongly downregulated and this was associated with cancer aggressiveness [62], albeit other studies have reported that moesin contributes to pathological state [63,64] and may be linked to metastasis [65]. PAD inhibitors were also found to modulate prohibitin (PHB), which is a multifaceted protein with key roles in mitochondrial housekeeping and tumourigenesis [66,67,68]. In pancreatic cancer, PHB was identified as a pro-tumour marker and negatively correlated with survival [69]. The assessment of proteins involved in cancer progression and invasion, as well as mitochondrial function, may be of considerable relevance, both with respect to PAD inhibitor-mediated changes in total protein levels and with respect to their post-translational deimination, as this may affect protein structure, function, and protein–protein interactions [6,7]. Effects of PAD inhibitor treatment on histone H3 deimination has also been assessed in cancers, which may be of importance as post-translational modifications of histones, including deimination, have been found to be players in cancers as well as in various inflammatory diseases and injury. For example, histone deimination was found to be reduced in response to Cl-amidine treatment in GBM [15] and reduced levels were also found in CNS injury following Cl-amidine treatment [70,71,72]. Histone citrullination is thought to mainly be modulated by PAD4, which has a classic nuclear localisation signal, although other PAD forms, including PAD2 and PAD3, were also found to localise to the nucleus and be able to deiminate histones, including H2A, H2B and H4 [13,14].

As current knowledge on PADs in PDAC is limited, this study aimed at assessing the effects of pan-PAD inhibitor Cl-amidine, as well as PAD2, PAD3, and PAD4 isozyme-specific inhibitors on PDAC cell invasion, on moesin and prohibitin protein levels, as well as putative effects on histone H3 deimination in two PDAC cell lines. Furthermore, modulatory effects of the PAD inhibitors on EV signatures were assessed, including pro- and anti-oncogenic miR EV cargo.

## 2. Results

### 2.1. PAD Isozymes Are Differently Expressed in PDAC Cells

**Figure 1:**
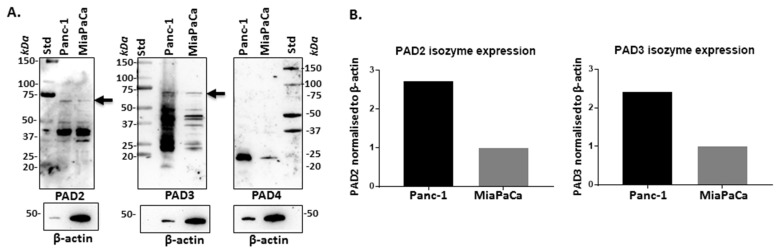
Peptidylarginine deiminase (PAD) isozyme expression in Panc-1 and MiaPaCa-2 cells. (**A**) PAD2 and PAD3 isoforms are detected at expected 70–75 kDa size range (arrows), although lower molecular weight bands are also observed. PAD4 isoform does not show positive at the expected size of 75 kDa, only a band at 25 kDa is observed. (**B**) PAD2 and PAD3 protein levels, normalised to internal beta-actin control, indicating that both isoforms are higher expressed in Panc-1 cells, compared with MiaPaCa-2 cells.

Assessing PAD2, 3, and 4 isozyme-specific antibodies on protein extracts from Panc-1 and MiaPaCa-2 cells showed bands at expected size for PAD2 and PAD3 (70–75 kDa size), while additional lower molecular weight bands were also observed. The PAD4 antibody showed only a band at low molecular weight (25 kDa), while no reaction was seen in either cell line at an expected 70–75 kDa size for PAD4 (Figure 1A), or a similar pattern to the other two PAD isozyme-specific antibodies. Quantifying the protein expression against internal loading control (beta-actin) further showed that PAD2 and PAD3 were higher expressed in Panc-1 cells, compared with MiaPaCa-2 cells (2.6- and 2.4-fold for PAD2 and PAD3, respectively) (Figure 1B).

### 2.2. Pan-PAD and PAD Isozyme-Specific Inhibitors Differently Modulate EV Release in Pancreatic Cancer Cells Following 1 h Treatment

**Figure 2:**
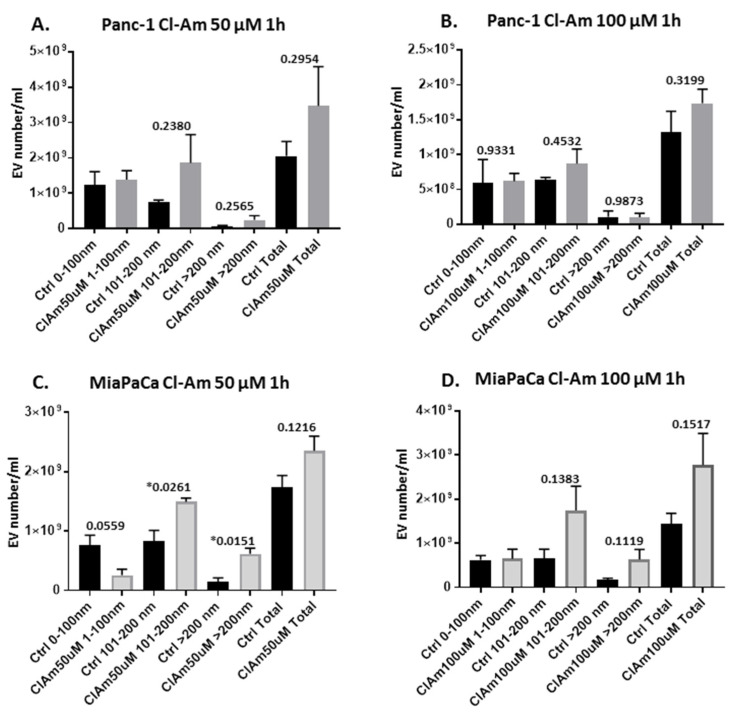
Pan-PAD inhibitor (Cl-amidine) treatment shows varying effects on the two pancreatic ductal adenocarcinoma (PDAC) cell lines under study with respect to cellular extracellular vesicle (EV) release. (**A**) Effects of Cl-amidine (50 μm 1 h treatment) on EV release from Panc-1 cells. (**B**) Effects of Cl-amidine (100 μm 1 h treatment) on EV release from Panc-1 cells. (**C**) Effects of Cl-amidine (50 μm 1 h treatment) on EV release from MiaPaCa-2 cells. (**D**) Effects of Cl-amidine (100 μm 1 h treatment) on EV release from MiaPaCa-2 cells. For each set of histograms, Cl-amidine (50 or 100 μm)-treated and control (PBS)-treated cells were run under the same experimental conditions, respectively. Exact *p*-values are indicated (* highlights significance at *p* ≤ 0.05), error bars show SD; *n* = 3 biological replicates for all).

A considerable difference was observed on EV release profiles following treatment with pan-PAD inhibitor Cl-amidine, compared with the PAD isozyme-specific inhibitors. Cl-amidine somewhat (albeit non-significantly) increased total EV release in both cell lines at both concentrations tested (50 and 100 μm) and had varying effect on different EV subpopulations, showing some reduction on EVs in the 0–100 nm size range (small EVs) in MiaPaCa-2 cells at 50 μm concentration (Figure 2).

**Figure 3:**
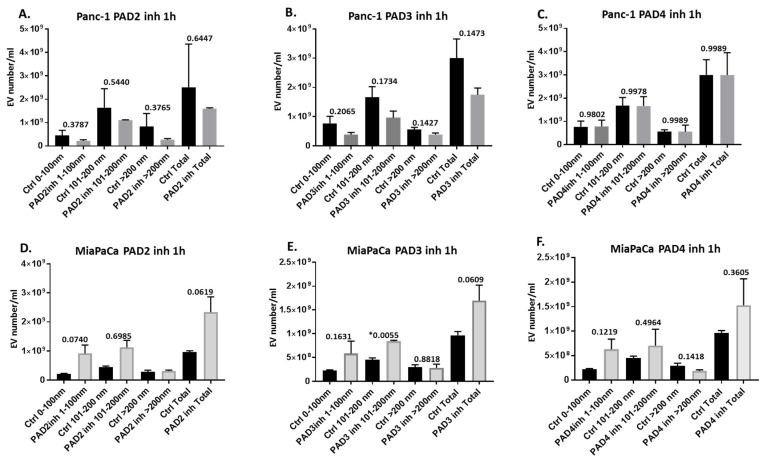
PAD2, PAD3, and PAD4 inhibitor treatments show PDAC cell line differing effects on cellular EV release. (**A**–**C**) Effects EV release from Panc-1 cells following 1 h treatment with: (**A**) PAD2 inhibitor, (**B**) PAD3 inhibitor, (**C**) PAD4 inhibitor. (**D**–**F**) Effects of PAD2 inhibitor on EV release from MiaPaCa-2 cells following 1 h treatment with: (**D**) PAD2 inhibitor, (**E**) PAD3 inhibitor, (**F**) PAD4 inhibitor. For each set of histograms, respectively, the PAD isozyme-specific inhibitor-treated and control-treated cells were run under the same experimental conditions. Exact *p*-values are indicated (* highlights significance at *p* ≤ 0.05), error bars show SD; *n* = 3 biological replicates for all.

**Figure 4:**
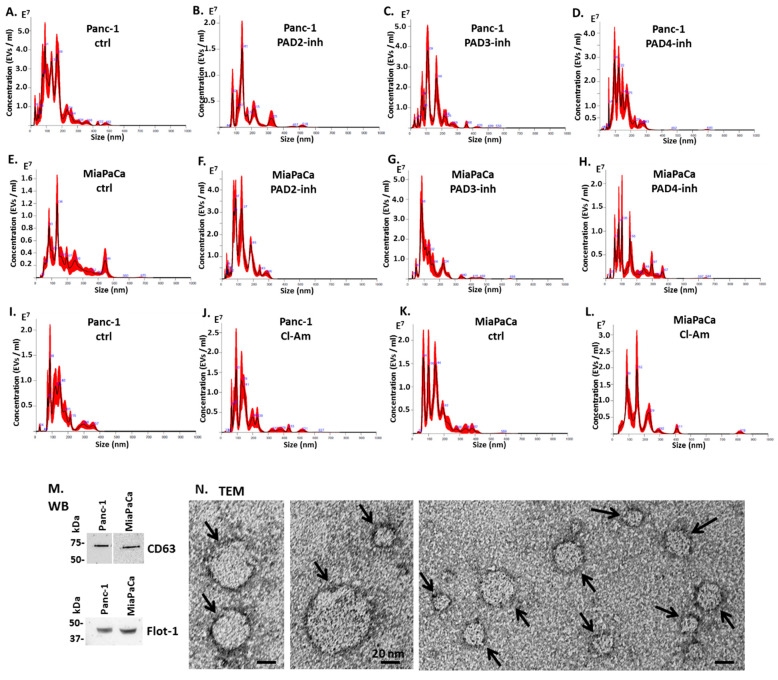
Nanoparticle tracking analysis (NTA) size distribution profiles of EVs released from Panc-1 and MiaPaCa-2 cells following PAD inhibitor treatment for 1 h and EV characterisation by Western blotting (WB) and transmission electron microscopy (TEM). (**A–D**) Representative NTA profiles of Panc-1 cells following 1 h PAD inhibitor treatment: (**A**) Control DMSO-treated cells; (**B**) PAD2 inhibitor-treated cells; (**C**) PAD3 inhibitor-treated cells; (**D**) PAD4 inhibitor-treated cells; Representative NTA profiles of MiaPaCa-2 cells following 1 h PAD inhibitor treatment (**E–H**): (**E**) control DMSO-treated cells; (**F**) PAD2 inhibitor-treated cells; (**G**) PAD3 inhibitor-treated cells; (**H**) PAD4 inhibitor-treated cells. (**I–L**) show representative NTA profiles of EVs released from pan-PAD inhibitor (Cl-amidine)-treated cells: (**I**) Control PBS-treated Panc-1 cells; (**J**) Cl-am (100 μm)-treated Panc-1 cells; (**K**) Control PBS-treated MiaPaCa-2 cells; (**L**) Cl-am (100 μm)-treated MiaPaCa-2 cells. (**M**) Western blotting analysis (WB) showing that EVs isolated from Panc-1 and MiaPaCa-2 cells are positive for the EV-specific markers CD63 and Flot-1. (**N**) Transmission electron microscopy (TEM) images showing characteristic EV morphology (arrows) for EVs isolated from PDAC cells under standard conditions; the scale bar represents 20 nm in all TEM images. In the NTA curves (**A–L**) the black line represents the mean of the five repetitive readings per individual sample and the red line represents standard error (+/−) between those same five readings per sample. Each treatment group was measured in three biological replicates.

When assessing the PAD isozyme-specific inhibitors, PAD2 inhibitor showed some reducing effects on total EV release in Panc-1 cells and on all sub-populations (albeit non-significant) (Figure 3A), while the opposite was observed for MiaPaCa-2, with increased EV release, although this was also non-significant (Figure 3D). PAD3 inhibitor reduced EV release in Panc-1 cells, including all sub-populations (albeit non-significant) (Figure 3B), but increased EV release somewhat (non-significant) from MiaPaCa-2 cells (Figure 3E). PAD4 inhibitor had no effects on EV release from Panc-1 cells (Figure 3C), but increased total EV release and small and medium-sized EV subpopulations, while it reduced release of larger EVs somewhat in MiaPaCa-2 cells (albeit all non-significant) (Figure 3F). Figure 4 shows representative nanoparticle tracking analysis (NTA) profiles for EV size distribution of EVs released from Panc-1 and MiaPaCa-2 control and PAD inhibitor treated cells (Figure 4A–L), alongside characterisation of EVs by Western blotting using the EV-specific markers CD63 and Flot-1 (Figure 4M). Morphology of EVs was verified by transmission electron microscopy (TEM) (Figure 4N).

**Figure 5:**
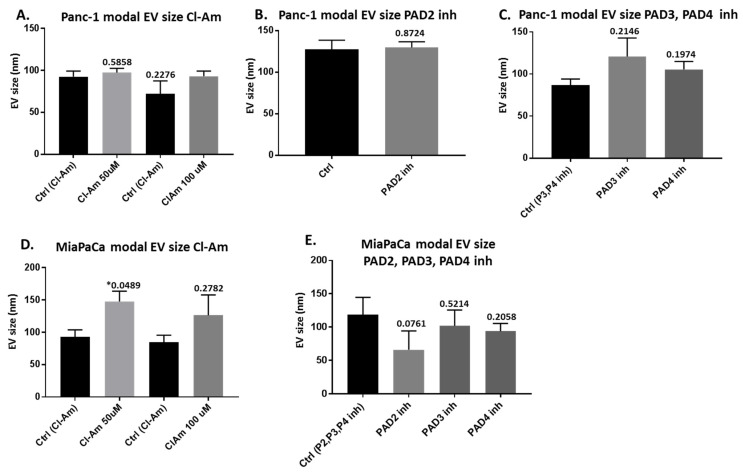
Modal EV size profiles from PDAC cells following 1 h treatment with pan-PAD and PAD isozyme-specific inhibitors. (**A**) Modal size of EVs released from Panc-1 cells following Cl-amidine treatment (50 and 100 μm respectively), compared with control (PBS)-treated cells (**B**) Modal size of EVs released from Panc-1 cells following PAD2 inhibitor treatment, compared with control (DMSO)-treated cells. (**C**) Modal size of EVs released from Panc-1 cells following PAD3 and PAD4 inhibitor treatment, compared with control (DMSO)-treated cells. (**D**) Modal size of EVs released from MiaPaCa-2 cells following Cl-amidine treatment (50 and 100 μm, respectively), compared with control (PBS)-treated cells. (**E**) Modal size of EVs released from MiaPaCa-2 cells following PAD2, PAD3, and PAD4 inhibitor treatment, compared with control (DMSO)-treated cells. The histograms show treatments run together, respectively. Exact *p*-values are indicated (* highlights significance at *p* ≤ 0.05), error bars show SD (*n* = 3 biological replicates for all).

EV modal size was overall not significantly affected following 1 h PAD inhibitor treatment although in MiaPaCa-2 cells Cl-amidine significantly increased modal size following 50 μm treatment; this trend was also seen after 100 μm treatment for both Panc-1 and MiaPaCa-2, although not significant. PAD2 inhibitor treatment had no effect on EV modal size of Panc-1 cells, but reduced modal size of EVs from MiaPaCa-2 cells somewhat (albeit non-significant). PAD3 inhibitor resulted in some increase in modal size of Panc-1-derived EVs (albeit non-significant), while PAD4 inhibitor had no significant effects on EV modal size in either cell line (Figure 5, exact p-values are indicated).

### 2.3. MicroRNA EV Cargo Is Differently Modulated in Response to 1 h PAD Inhibitor Treatments in PDAC Cells

**Figure 6:**
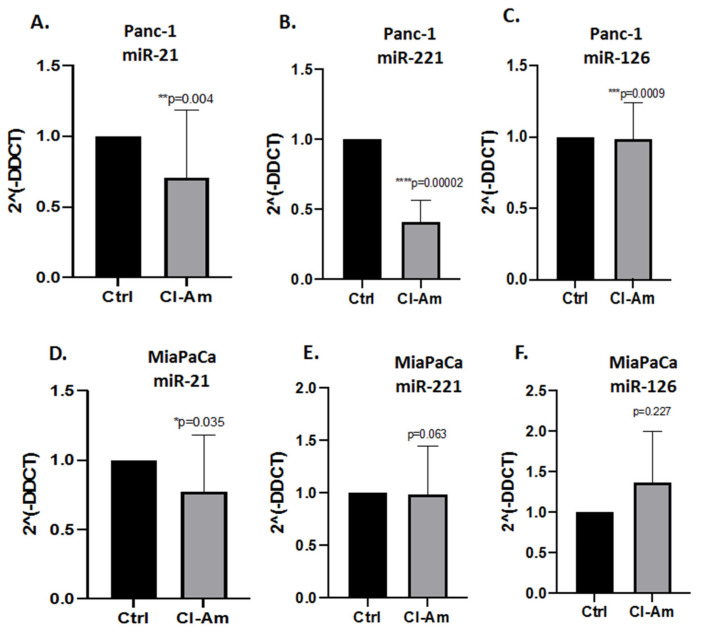
PAD inhibitor (Cl-amidine and isozyme-specific) mediated effects on EV microRNA cargo in PDAC cells. (**A**–**C)**: Effects of Cl-amidine (1 h treatment at 100 µm) in Panc-1 cell-derived EVs on: (**A**) miR-21; (**B**) miR-221; (**C**) miR-126. (**E**–**F**): Effects of Cl-amidine on MiaPaCa-2 cell-derived EVs (1 h treatment at 100 µm) for: (**D**) miR-21; (**E**) miR-221; (**F**) miR-126. Exact *p*-values are indicated (* *p* ≤ 0.05; ** *p* ≤ 0.01; *** *p* ≤ 0.001; **** *p* ≤ 0.0001); error bars indicate SD.

**Figure 7:**
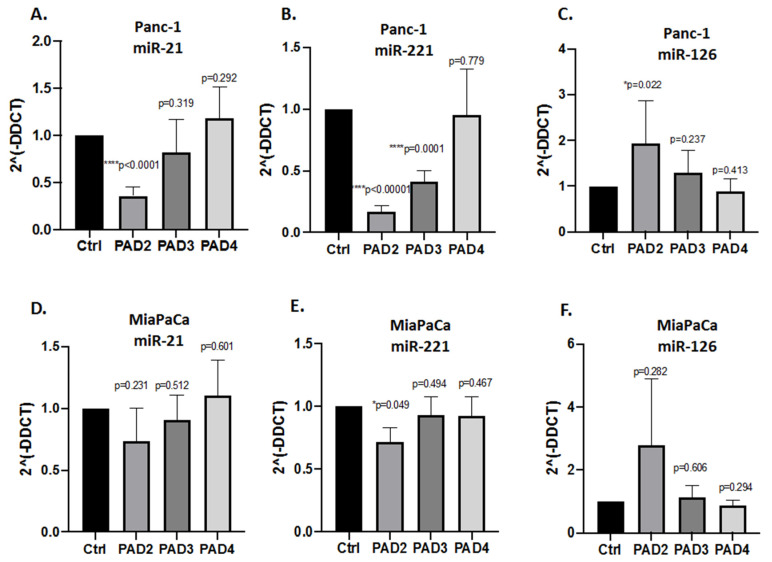
PAD isozyme-specific inhibitor (PAD2, PAD3, PAD4 inhibitor) mediated effects on EV miR cargo in PDAC cells. (**A–C**): Effects on Panc-1 cell-derived EVs for: (**A**) miR21; (**B**) miR221; (**C**) miR126. (**E–F**): Effects on MiaPaCa-2 cell-derived EVs for: (**D**) miR-21; (**E**) miR-221; (**F**) miR-126. Exact *p*-values are indicated (* *p* ≤ 0.05; *** *p* ≤ 0.001; **** *p* ≤ 0.0001); error bars indicate SD.

When assessing EV cargo for pro-cancerous-related miRs (miR-21, miR-221) respectively, some significant expression changes were observed in response to the different PAD inhibitors (Figure 6 and Figure 7). Pan-PAD inhibitor Cl-amidine (100 µm treatment for 1 h) significantly reduced miR-21 in Panc-1 cells (Figure 6A), had no significant effects on miR221 EV cargo (Figure 6B), and reduced miR-126 somewhat (Figure 6C). In MiaPaCa-2 cells, Cl-amidine did not have significant effects on levels of the different miRs assessed (Figure 6D–F), but did increase miR-126 somewhat, albeit not significantly.

Following PAD isozyme-specific inhibitor treatment for 1 h, PAD2 and PAD3 inhibitors reduced both miR-21 and miR-221 significantly in Panc-1 cell-derived EVs, while PAD4 inhibitor had no significant effects (Figure 7A,B). miR-126 was elevated following PAD2 and PAD3 inhibitor treatment (significantly for PAD2 inhibitor), while PAD4 inhibitor had no effects (Figure 7C). In MiaPaCa-2 cells, none of the isozyme-specific inhibitors had significant effects on miR-21 cell-derived EV cargo, albeit some reduction as observed with PAD2 inhibitor (Figure 7D) and significant reduction in miR-221 EV content was observed for PAD2 inhibitor. miR-126 was elevated in response to PAD2 inhibitor treatment (albeit not reaching statistical significance due to variation in the sample), while PAD3 and PAD4 inhibitors had no effect on miR-126 levels in MiaPaCa-2 cells (Figure 7F).

### 2.4. PAD Inhibitors Differently Affect Moesin, PHB, and Deiminated Histone H3 Protein Levels in Panc-1 Cells, Following 1 h Treatment

**Figure 8:**
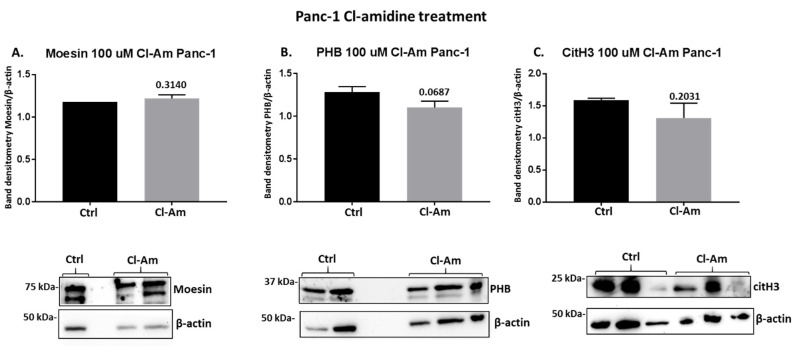
Changes in moesin, prohibitin, and deiminated histone H3 (citH3) protein levels in Panc-1 cells following 1 h Cl-amidine treatment. The histograms show protein levels normalised against the internal loading control (β-actin) in treated versus control-treated cells as follows: (**A**) Moesin levels are not significantly changed; (**B**) Prohibitin (PHB) levels are slightly reduced following Cl-Am treatment; (**C**) citH3 levels are slightly reduced following Cl-Am treatment. Exact *p*-values are shown (significance at *p* ≤ 0.05 was not reached), error bars show SD (*n* = 3 biological replicates for all, representative Western blots are shown beneath the respective histograms).

**Figure 9:**
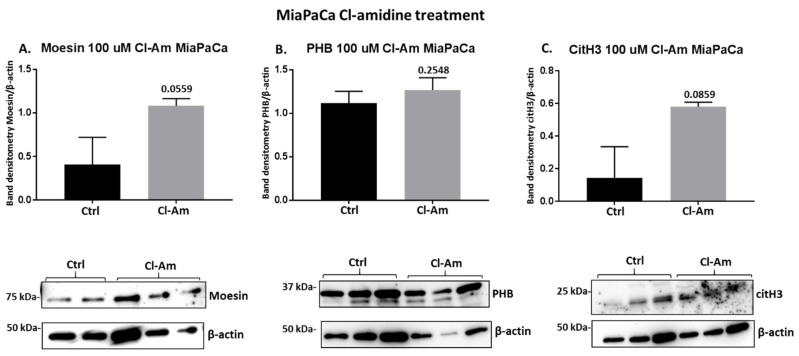
Changes in moesin, prohibitin, and citH3 protein levels in MiaPaCa-2 cells following 1 h Cl-amidine treatment. The histograms show protein levels normalised against the internal loading control (β-actin) in treated versus control-treated cells as follows: (**A**) Moesin levels are increased (not reaching significance) following Cl-amidine treatment; (**B**) Prohibitin (PHB) levels are not significantly affected following Cl-Am treatment; (**C**) citH3 levels are increased (not reaching significance) following Cl-Am treatment. Exact *p*-values are shown (significance at *p* ≤ 0.05, which was not reached), error bars show SD (*n* = 3 biological replicates for all, representative Western blots are shown beneath the respective histograms).

**Figure 10:**
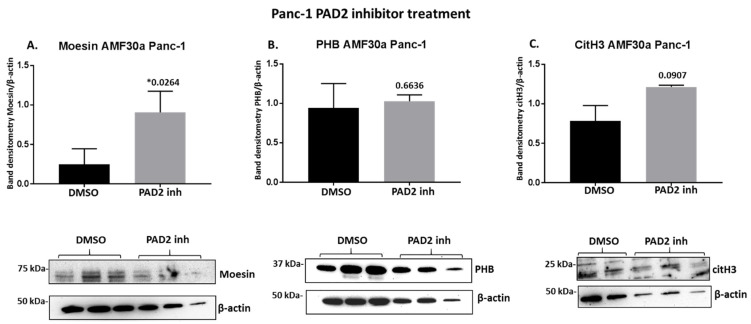
PAD2 inhibitor treatment (1 h) effects on moesin, prohibitin, and citH3 levels in Panc-1 cells. (**A**) PAD2 inhibitor treatment significantly increased Moesin levels in Panc-1 cells. (**B**) No significant effect was observed on PHB levels following 1 h PAD2 inhibitor treatment in Panc-1 cells. (**C**) Histone H3 deimination (citH3) was somewhat increased following 1 h PAD2 inhibitor treatment, but not reaching significance. The error bars show SD, exact *p*-levels are shown (* highlights significance at *p* ≤ 0.05), and representative Western blots are shown for all protein assessments. The histograms are based on *n* = 3 per treatment.

Following 1 h treatment with pan-PAD inhibitor (Cl-amidine) and the PAD2 isozyme-specific inhibitor (AMF30a), respectively, the protein levels of moesin, PHB, and deiminated histone H3 (citH3) were assessed by Western blotting (Figure 8). In Panc-1 cells, Cl-amidine did not affect moesin levels (Figure 8A), had some reducing (albeit non-significant) effect on PHB levels (Figure 8B) and some reducing (albeit not significant) effect on citH3 levels (Figure 8A). In MiaPaCa-2 cells, moesin levels were increased following Cl-amidine treatment (albeit not reaching significance, *p* = 0.056) (Figure 9A), PHB protein levels were not significantly changed (Figure 9B), while citH3 levels were increased (albeit non-significant) (Figure 9C). In addition, PAD2 inhibitor treatment changes in these proteins were further assessed in the Panc-1 cell line only, as PAD2 inhibitor showed most effects on EV signatures, and Panc-1 is considered the more aggressive PDAC cell line. In response to 1 h PAD2 inhibitor treatment, a strong significant increase was observed in moesin protein levels (Figure 10A), while no effect was observed on PHB levels (Figure 10B), and citH3 levels were somewhat (albeit not significantly) increased (Figure 10C).

### 2.5. PAD Isozyme-Specific Inhibitors Differently Affect Panc-1 Cell Invasion, Independent of Cell Proliferation

**Figure 11:**
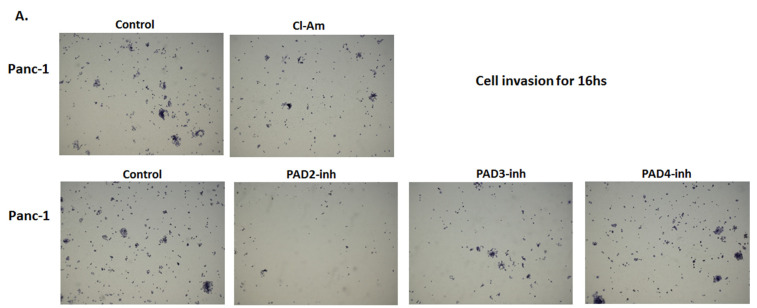

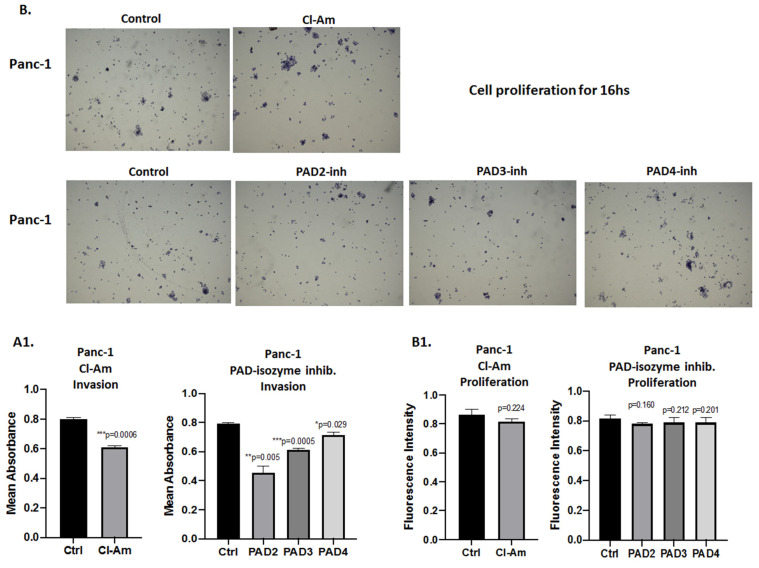
Cell invasion is differently affected by Cl-amidine and PAD2, 3, 4 isozyme-specific inhibitors in Panc-1 cells. (**A**) Cell invasion: Representative images (taken at 10× magnification) for cell invasion of Panc-1 cells following 16 h treatment with Cl-amidine and the three PAD isozyme-specific inhibitors, compared to control (PBS or DMSO) treated cells are shown; cells are stained with crystal violet. (**A1**). The corresponding histogram for the MTT assay for Panc-1 cells, following the 16 h experiment in the presence of Cl-amidine and the three PAD isozyme-specific inhibitors, compared with control, respectively. (**B**) Cell proliferation: Representative images (taken at 10× magnification) for cell proliferation of Panc-1 cells following 16 h treatment with Cl-amidine and the three PAD isozyme-specific inhibitors, compared to control (PBS or DMSO) treated cells; cells are stained with crystal violet. (**B1**). The corresponding histogram for Panc-1 cell proliferation following 16 h treatment with Cl-amidine and the three PAD isozyme-specific inhibitors. Exact *p*-values are indicated (statistically significant differences are highlighted as * *p* < 0.05; ** *p* < 0.01 and *** *p* < 0.001).) and error bars show SD (*n* = 3 biological and 3 technical replicates for all).

**Figure 12:**
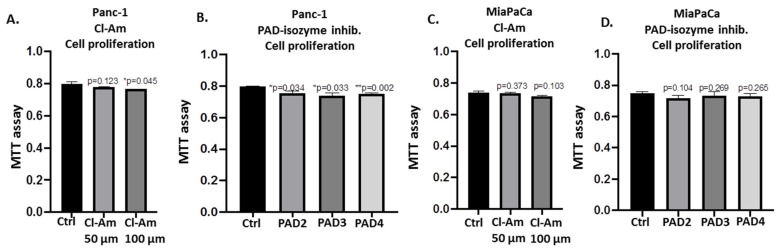
Cellular proliferation following Cl-amidine and/or PAD2, 3, 4 isozyme-specific inhibitor treatment in Panc-1 and MiaPaCa-2 cells. MTT assay showing cell proliferation after 1 h treatment with Cl-Am (50 and 100 μm) shows little effect on either Panc-1 (**A**) or MiaPaCa-2 cells (**C**), albeit in some cases statistical significance is reached. Similarly, PAD2 (5 μm), PAD3 (10 μm), and PAD4 (10 μm) inhibitors did not show marked changes on cell proliferation in Panc-1 cells (**B**) or MiaPaCa-2 cells (**D**) following 1 h treatment. Exact *p*-values are indicated (* *p* ≤ 0.05; ** *p* ≤ 0.01).

The effect of pan-PAD inhibitor Cl-amidine and the isozyme-specific PAD2, PAD3, and PAD4 inhibitors, respectively, was assessed on the cell invasiveness of Panc-1 cells using Boyden chambers with Matrigel (Figure 11A). Panc-1 cells demonstrated noticeable invasion over 16 h (Figure 11A control), and incubation for 16 h with PAD2 and PAD3 inhibitors resulted in the most significant suppression of invasiveness by 42.7% for PAD2 inhibitor (*p* = 0.005) and 22.6% reduction for PAD3 inhibitor (*p* = 0.0005), respectively, while less effect was observed following treatment with Cl-amidine (23.3% reduction; *p* = 0.0006), and least effect was seen following treatment with the PAD4 inhibitor (9.8% reduction; *p* = 0.029) (Figure 11A; n = 3 per treatment; see histograms for quantitative assessment and representative figures of the cell invasion experiments). It must be noted, though, that PAD4 inhibitor did show some effects on cell invasiveness and therefore, while by Western blotting PAD4 was not detected at the correct molecular size, the expression and function of PAD4 in PDAC cells requires further investigation. Cell proliferation was also assessed for Panc-1 cells, following 16 h treatment with the different PAD inhibitors and showed negligible, confirming that the inhibitors did not affect proliferation. Cl-amidine treatment resulted in 5.8% reduction in cell proliferation, PAD2 inhibitor in 4.1% reduction in cell proliferation, PAD3 inhibitor in 3.1% reduction in cell proliferation, and PAD4 inhibitor in 3.1% reduction in cell proliferation (Figure 11B; see histograms for quantitative assessment and representative figures of the cell invasion experiments). Cell proliferation was also assessed for both cell lines for all PAD inhibitors used for a 1 h period, confirming no marked changes in cell proliferation for the concentrations used in either cell line (Figure 12).

## 3. Discussion

This study assessed roles for PADs and PAD regulatory effects in two PDAC cell lines. Roles for PADs have previously been studied in relation to a range of cancers, with respect to epigenetic regulation, cancer invasion, and EV release. Hitherto though, knowledge on PADs in PDAC is limited.

We established that PAD2 followed by PAD3 were the dominant isoforms in the PDAC cell lines under study (Panc-1 and MiaPaCa-2), while PAD4 protein was not detected at the expected molecular size using Western blotting. This was further reflected in effects of PAD isozyme-specific inhibitors on PDAC cell invasion, showing the highest effects for PAD2 inhibitor, followed by PAD3 inhibitor, while PAD4 inhibitor was far less effective, compared with the other PAD inhibitors. It must be noted, though, that some effects were observed of PAD4 inhibitor, and while PAD4 was not detected at the expected molecular weight band in these cell lines, the expression and roles for PAD4 in PDAC will require further investigation. Pan-PAD inhibitor Cl-amidine, which has specificity against PAD1, PAD3, and PAD4 [73,74], also showed less effects compared with the PAD2-specific inhibitor. While some variability was observed in cell proliferation following treatment with the different PAD inhibitors, comparing 1 and 16 h treatment times, this was below 6% in all cases.

This is the first study to assess the three different PAD isozymes in PDAC, while previous studies have indeed pointed to roles for citrullination/deimination in PDAC, including via deimination of specific target proteins (ENO1, HSP60, KRT8, and TUBB) [75]. Roles for neutrophil extracellular trap formation (NETosis) have also been implied [76], which can be partly PAD-driven and NETosis is commonly assessed using citH3 staining. Furthermore, using mouse models of PDAC, it was reported that circulating tumour-derived EVs and citH3 levels are elevated [77]. In addition, tumour-infiltrating NETs, as assessed by citH3 staining, were also shown to predict poor postsurgical survival of patients with PDAC [78]. Previously, deiminated α-enolase was identified as a target for anti-cancer immunity, including in PDAC murine models [79]. Interestingly, a patient diagnosed with fatal metastatic pancreatic cancer showed anti-cyclic citrullinated peptide antibodies that were linked to cancer polyarthritis [80]. It was also hypothesised that the deiminating activity of PAD and PAD homologues (ADI) in oral bacteria can contribute to pancreatic cancer [81].

Due to roles for EVs in cancer progression and previous observations for PAD inhibitor modulating effects on EV biogenesis and EV cargo, we assessed effects of PAD isozyme-inhibitor treatment on changes in EV signatures in Panc-1 and MiaPaCa-2 cell lines, including pro- and anti-oncogenic EV miR cargo (miR-21, miR221, and miR-126). We found that EV signatures were differently modulated in response to the PAD isozyme-specific inhibitors, including some effects observed on EV subpopulations and EV miR cargo. Overall, the strongest effects observed on EV signature modulation were for PAD2 inhibitor in Panc-1 cells, followed by PAD3 inhibitor. These PAD inhibitors reduced pro-oncogenic miR-21 and miR-221 EV cargo and elevated anti-oncogenic miR-126. The effects of PAD inhibitors on miR signatures is of considerable interest as several signalling pathways are associated with miRs in PDAC progression [82], including for the three miRs under study here.

miR-21 is one of the main onco-miRs in a range of cancers, and its overexpression is associated with an elevated proliferation and invasion of PDAC cells [83]. Upregulation of miR-21 and downregulation of miR-126 are considered to contribute to PDAC progression through post-transcriptional upregulation of KRAS [36]. It has been suggested that miR-21 is involved in oncogenic RAS-induced cell proliferation [38] and indirectly targets KRAS, while downregulation of miR-126 directly targets KRAS signalling pathway and controls KRAS protein translation [39]. Also, upregulation of miR-21 targets PTEN, which further suppresses PI3K-AKT-mTOR signalling pathway, and this can lead to inhibition of cell cycle arrest, apoptosis, and gemcitabine sensitivity [84].

miR-221 plays a significant role in platelet-derived growth factor (PDGF)-mediated EMT phenotype, migration, metastasis, and uncontrolled proliferation of PDAC cells [59]. An additional target gene, which is associated with miR-221 expression levels, is CDKN1B [85], which inhibits cell cycle through the modulation of cell proliferation, cell motility, and apoptosis [86]. Specifically, the overexpression of miR-221 can lead to the loss of expression of CDKNs, which are associated with unfavourable prognosis of PDAC [87].

miR-126, contrary to miR-21 and miR-221, can act as a tumour suppressor in several carcinomas such as lung, gastric, breast and PDAC through the inhibition of epidermal growth-factor-like domain 7 (EGFL7), Crk, and SLC7A5 [88]. Furthermore, altered expression of this miR can have as a consequence cellular migration and invasion through the inhibition of ADAM metallopeptidase domain 9 (ADAM9) target gene, which is commonly expressed in PDAC [89]. Aberrant expression of miR-126 in PDAC is associated with HER2 overexpression [40,41], which is related to more than 30% of PDAC cases [42], while direct correlation to survival rate has not been established [43] and the literature still remains controversial. Overall, the increase in the anti-oncogenic miR observed here in response to PAD inhibitor treatment points to anti-cancerous roles of PAD inhibitor application in PDAC.

We furthermore assessed effects of the PAD inhibitors on changes in histone H3 deimination (citH3), as well as in proteins relating to cell invasion (moesin) and mitochondrial housekeeping (PHB), all of which we have previously identified to be modulated by PAD inhibitor treatment in GBM cells [15,17]. As all three proteins are also involved in pancreatic cancer, alongside other cancers, changes in their cellular protein levels were assessed following pan-PAD inhibitor and PAD isozyme-specific inhibitor treatment. Furthermore, as Panc-1 cells showed higher levels of PAD protein than MiaPaCa-2, they were used to assess cell invasion capability in the presence of the pan-PAD (Cl-amidine) and the PAD2, 3, and 4 isozyme-specific inhibitors. The PAD2-specific inhibitor displayed the strongest effects on reducing Panc-1 cell invasion capability, which was accompanied by an increase in moesin expression. This effect on moesin levels may be protective in PDAC, as a previous study identified that moesin expression is low in pancreatic cancer, including PDAC, and that moesin knock-down increased migration, invasion, and metastasis [62]. Furthermore, some reduction, albeit not significant, was found in PHB levels following Cl-amidine treatment in Panc-1 cells, but not with the other inhibitors, and was also identified in GBM following PAD inhibitor treatment [15,17]. Previous studies have identified PHB to be elevated in PDAC, to negatively correlate with survival, and furthermore, siRNA of PHB resulted in decreased cell invasion [69]. PHB also plays important roles in ERK-driven pancreatic tumorigenesis [90]. Effects on citH3 were also assessed as histone deimination has been associated to a range of cancers, both for epigenetic regulation as well as in relation to NETosis, including pancreatic cancer and associated venous thrombosis [76]. Results for citH3 protein levels varied, with some reduction (but not significant) in Panc-1 cells following Cl-amidine treatment, while citH3 was elevated in MiaPaCa-2 cells. Also, following PAD2 inhibitor treatment, citH3 was elevated in Panc-1 cells. In addition to PAD4, both PAD2 and PAD3 have been localised and detected in the nucleus in spite of lacking a classic nuclear translocation site such as is found in PAD4 [70,91,92]. In cancer cells, PAD2, which is the most widely expressed isozyme in the body [93], was shown to deiminate histone H3 and play a role in gene regulation [91,94,95,96]. As PAD4 has been considered the main responsible isoform for citH3 generation, this warrants further investigation. Also, the difference observed between the two cell lines may be of some interest, showing cancer sub-type differences in response to the different PAD inhibitors. Previously, some reduced levels of citH3 were found in GBM following Cl-amidine treatment [15]. While our current study provides some pilot insights into putative roles for PAD inhibitors modulating citH3 in PDAC, it will be of great interest to further assess changes in citH3 in additional PDAC cell lines, by both Western blotting and immunocytochemistry, following treatment with the different PAD inhibitors.

The effect on elevated moesin levels following PAD2 inhibitor treatment observed here may be of some interest. Moesin is an ezrin-radixin-moesin (ERM) family member and involved in the regulation of cell adhesion, polarity, and migration [97]. Moesin has been associated with formation of filopodia, which are dynamic actin-rich membrane protrusions important for cell adhesion, membrane trafficking (including EV internalisation) [61], and therefore also of importance in cancer cell adhesion and invasion [98,99,100]. Increased moesin expression was related to metastasis and to advanced clinical stage in ER-positive breast cancer [101], while in higher grade GBM, moesin overexpression is also related to increased stem cell neurosphere formation [102,103]. In pancreatic cancer there are some contradictory findings regarding moesin expression. Studies have reported that moesin affects the progression of PC by activating MMP-7 and further promoting the release of TNF-α and IL-6 and decreasing the level of IL-10. The expression of moesin in PC tissues has close relations with the pathological stage of the disease, nerve infiltration, tumour location, and pain severity [64]. It was also reported, though, that moesin expression is reduced in PDAC and this is associated with pancreatic cancer aggressiveness, showing that moesin knock-down increased migration, invasion, and metastasis of pancreatic cancer and, furthermore, influenced pancreatic cancer extracellular matrix organisation [62]. Moreover, moesin-dependent cytoskeleton remodelling is associated with an anaplastic phenotype of pancreatic cancer [62]. Based on these findings, increasing moesin expression should be anti-oncogenic in pancreatic cancer, and would align with elevated moesin protein levels observed particularly with PAD2 inhibitor treatment in Panc-1 cells in the current study. In previous studies moesin was found to be reduced in response to PAD inhibitor treatment in GBM [17]. The roles of moesin may be cancer type-specific or also differ in cancer subtypes, while deimination of moesin may also play roles, and this will require further investigation.

While PAD2 inhibitor followed by PAD3 inhibitor had the most effects on reducing cancer cell invasion, elevating moesin expression, and modulating EV signatures in the PDAC cells in the current study, PAD4 inhibitor had negligible effects and pan-PAD inhibitor Cl-amidine was also less effective. This correlates with the differences observed in protein levels of the PAD isozymes in the cell lines and lack of positive signal for PAD4 at expected molecular size, also pointing to a negligible role for PAD4 in PDAC, while PAD2 was the most prominent isozyme. Some differences were furthermore observed between the two PDAC cell lines under study, pointing to stronger effects of PAD inhibitors in Panc-1 cells, which, interestingly, also showed substantial response to PAD3 inhibitor, which may correlate with previous observations that this cell line contains neuronal-like/stem-like properties [104].

Recent studies have indeed identified multifactorial roles for PAD2, PAD3, and PAD4 in cancer pathologies, depending on tumour type and cell lines [15,17,105,106,107,108,109]. PAD2 was found to play roles in gastric cancer and to have deleterious effects on tumour growth and metastasis in liver tumour cells [105]. Downregulation of PAD2 was on the other hand associated with colon cancer, while in normal colons PAD2 affects differentiation and can suppress proliferation of colonic epithelial cells [107,108]. PAD inhibitor Cl-amidine was shown to induce the upregulation of tumour suppressor miRs in colon cancer cells [110], as well as anti-oncogenic miR-126 in GBM [15]. Inhibition of PAD2 expression in breast cancer (MCF-7 cells) significantly decreased cell migration ability but did not affect cell proliferation and apoptosis [106], while use of Cl-amidine in MCF-7 cells reduced EV release and sensitised MCF-7cells to chemotherapy [12]. PAD4 also negatively regulates tumour invasiveness in breast cancer *in vitro* and *in vivo* models via citrullination of glycogen synthase kinase-3β (GSK3β) [109]. In GBM *in vitro* models, PAD2, 3, and 4 isozyme expression was shown to differ between GBM cell lines [15], which correlated to different effectivity of the PAD isozyme-specific inhibitors on invasion properties and EV signature modulation [17]. Therefore it may be relevant to assess PAD isozyme-selective inhibitors for intervention with regard to tumour type, and cancer subtypes. Furthermore, as we did not analyse effects of PAD inhibitors on apoptosis and potential alteration of the cell cycle in this study, this warrants further investigation.

It may be of interest that, compared with MiaPaCa-2 cells, stronger modulatory effects for the PAD inhibitors were observed in Panc-1 cells, which importantly also showed strong response to PAD3 inhibitor. PAD3 has been associated with neuronal stem cell properties [92] and found, for example, to be elevated in specific GBM cell lines, which may therefore also have stronger stem-like properties as GBM are known for stem-ness [15,17]. Importantly, previous PDAC in vitro studies reported that Panc-1 cells display most neuronal/stem-like properties [104], which may cause it to reflect a more aggressive form and also highlight possible roles for PAD3 in such stem-like cancers. Therefore it will be of great interest to further investigate a link between PAD3 and cancer stem-ness in a wider range of cancers and cancer cell lines, including PDAC. In the context of cancer evolution, transmissible cancer types in the animal kingdom have been shown to display evolutionary conserved immune evasion pathways and, interestingly, also neuronal properties [111,112]. Therefore, it may be speculated that roles for PAD isozymes in cancer evolution are of some interest and of putative importance for wide ranging cancer type-selective treatment.

In summary, our findings identify novel roles for PADs in PDAC and furthermore highlight roles for the different PAD isozymes in different cancer types, as well as cancer subtypes, and the potential for PAD isozyme-specific treatment to promote anti-oncogenic pathways in PDAC.

## 4. Materials and Methods

### 4.1. Pancreatic Cell Cultures and PAD Inhibitor Treatment

Panc-1 (ATCC^®^ CRL-1469™) and MiaPaCa-2 (ATCC^®^ CRL-x1420™) PDAC cells were cultured according to the recommendations of ATCC, using 75 cm^2^ flasks and complete Dulbecco’s Modified Eagle’s Medium (DMEM), with 10% foetal bovine serum (FBS) at 37 °C/5% CO_2_. The cells were split at 3–5 day intervals, depending on confluence. Cells were grown to 80% confluency in preparation for 1 h treatment with Cl-amidine (50 and 100 μm; Cayman Chemical, Michigan, USA), PAD2 (AMF30a, 5 μm [113]), PAD3 (Cl4-amidine, 10 μm [74]) and PAD4 (GSK199, 10 μm [114]) inhibitors, respectively, based on cell viability tests (see Section 4.2) and also based on previously published literature using these inhibitors [12,15,16,17,74,113,114]. The PAD inhibitors were dissolved in 0.001% DMSO (except Cl-amidine, which was dissolved in PBS) and DMSO (0.001%) or PBS-treated cells were used as controls, respectively. To assess effects on EV release, protein and miR expression, the cells were treated with the PAD inhibitors for 1 h, while cell proliferation was assessed at 1 h and 16 h, and for the invasion assays the treatment time was 16 h. For the isolation of Panc-1 and MiaPaCa-2 cell-derived EVs, the serum-containing medium was removed before application of the PAD inhibitors to avoid contamination of EVs from the FBS. Furthermore, before application of the medium containing the PAD inhibitors, the cells were washed in DPBS, and serum-free medium containing the respective PAD inhibitors were added for 1 h. DMSO or PBS was used as corresponding control treatment. Following 1 h incubation, the medium containing the EVs was removed from all treatment flasks, and the EVs were isolated as described in Section 4.4. For preparation of cell protein extracts for Western blotting (Section 4.7), the cells were trypsinised before further processing.

### 4.2. Cell Viability Assays Following PAD Inhibitor Treatment

The viability of Panc-1 and MiaPaCa-2 cells was assessed following 1 h incubation with pan-PAD inhibitor Cl-amidine (50 and 100 μm), PAD2 (AMF30a, 5 μm), PAD3 (Cl4-amidine, 10 μm), and PAD4 (GSK199, 10 μm) isozyme-specific inhibitors, respectively, compared to PBS or DMSO control-treated cells. The procedure was similar to that previously described [17]. Cells were seeded at a density of 1 × 10^4^ onto a 96-well plate (Nunc, Denmark) for 2–3 days. Cells were treated with either medium containing only PBS or DMSO, compared with Cl-amidine, PAD2, PAD3, and PAD4 inhibitors, respectively (at the same concentrations as in Section 4.1), at 37 °C, 5% CO_2_ for 1 h. To determine the effect of the PAD inhibitors on cell proliferation, MTT (3-(4,5-dimethylthiazol-2-yl)-2,5-diphenyl tetrazolium bromide) assay (Abcam, U.K.) was performed. A CLARIOstar plate reader (BMG Labtech, U.K.) was used to measure absorbance at 540–590 nm and normalised to the control. The experiments were performed in three biological and three technical repeats. Based on outcomes of this assay, further experiments (except initial EV modulation effects) involving Cl-amidine were carried out using Cl-amidine at 100 µm concentration.

### 4.3. Modulation of EV Release Using Cl-Amidine, PAD2, PAD3, and PAD4 Isozyme-Specific Inhibitors Following 1 h Treatment

The effect of Cl-amidine (50 and 100 μm), PAD2 (AMF30a; 5 μm), PAD3 (Cl4-amidine; 10 μm), and PAD4 (GSK199; 10 μm) isozyme-specific inhibitors was assessed on EV release from Panc-1 and MiaPaCa-2 cells, following 1 h treatment according to previously described methods [17]. Panc-1 and MiaPaCa-2 cells were cultured and maintained in triplicates in T75 flasks, in the presence of pre-warmed culture medium (DMEM, supplemented with 10% FBS; Sigma-Aldrich, U.K.), according to the recommendations of ATCC. Both cell lines were grown to 80% confluency per T75 flask, whereafter the cells were split in culture medium (5 mL per T25 flask of pre-warmed DMEM, supplemented with 10% FBS; Sigma-Aldrich, U.K.). Each experiment was carried out when the cells in the flasks had reached 70–80% confluency. For EV isolation, treatment with Cl-amidine or the PAD isozyme-specific inhibitors and PBS or 0.001% DMSO, respectively, was carried out in biological triplicate per treatment as follows: Before PAD inhibitor application (or PBS/DMSO as controls), the serum-containing medium was removed from the T25 flasks, to avoid contamination of EVs from the FBS in the medium, and the cells were washed three times with pre-warmed Dulbecco’s PBS (DPBS). Then fresh pre-warmed serum- and EV-free DMEM, containing either the PAD inhibitors or control treatment (PBS, DMSO) (using 5 mL medium per T25 flask) were added for 1 h at 37 °C/5% CO_2_. Thereafter, the EV-containing media were collected, cell debris removed by centrifugation at 200× *g* for 10 min and the EVs then isolated from the remaining supernatant (Section 4.4).

### 4.4. EV Isolation and Quantification by Nanoparticle Tracking Analysis

EV isolation was carried out according to previously established protocols [15,17] and according to the recommendations of the International Society of Extracellular Vesicle Research (ISEV) [115]. Differential centrifugation of the cell culture supernatants (5 mL collected from each flask) was carried out as follows: Supernatants were first centrifuged at 4000× *g* for 30 min at 4 °C for removal of cell debris, and the remaining supernatant ultracentrifuged for 1 h/4 °C at 100,000× *g* for EV enrichment. The supernatant was discarded, the EV-enriched pellets resuspended in ice-cold DPBS, and the EVs ultracentrifuged again for 1 h/4 °C at 100,000× *g*. The final EV pellet was resuspended in 100 μL sterile DPBS. Nanoparticle tracking analysis (NTA) using the NS300 Nanosight (Malvern Panalytical Ltd., Malvern, U.K.) was carried out, using a 405 nm diode laser and a sCMOS camera, for EV quantification. For recording on the NTA, the samples were diluted 1:100 in sterile-filtered EV-free DPBS, keeping the particle number per field of view at 30–50 and the minimum concentration of samples at 5 × 10^7^ particles/mL. The camera settings were according to the manufacturer’s instructions (Malvern Panalytical Ltd., Malvern, U.K.). Four 90 s videos were recorded per sample and averaged to obtain replicate histograms. Each experiment was repeated in three biological replicates.

### 4.5. EV Characterisation by Transmission Electron Microscopy

TEM was carried out according to previously described methods [15,17]. In brief, EVs from Panc-1 and MiaPaCa-2 cells were resuspended in 100 mM sodium cacodylate buffer (pH 7.4) and placed on a glow discharged grid with carbon support film. The grid was then placed in 2.5% glutaraldehyde in 100 mM sodium cacodylate buffer (pH 7.4), followed by washing and staining with 2% aqueous Uranyl Acetate (Sigma-Aldrich). EVs were visualised using a JEOL JEM 1400 transmission electron microscope (JEOL, Japan), at a magnification of 30,000 to 60,000 and operated at 80 kV at a magnification of 30,000 to 60,000. An AMT XR60 CCD camera (Deben, U.K.) was used for recording of digital images.

### 4.6. Analysis of MicroRNAs miR-21, miR-126, and miR-221 in EV Cargo Following 1 h PAD Inhibitor Treatment

MiR cargo was assessed in Panc-1 and MiaPaCa-2 cell-derived EVs, isolated from PAD inhibitor-treated and control-treated T25 flasks following 1 h incubation, as described above and according to previously described methods [17]. The EVs were processed for RNA isolation, followed by cDNA translation and assessment for the relative expression of miR-21, miR-126, and miR-221. RNA extraction was carried out using Trizol (Sigma, U.K.), while RNA concentration and purity were measured by NanoDrop Spectrophotometry at 260 nm and 280 nm absorbance. Reverse transcription of RNA to cDNA was carried out using the qScript microRNA cDNA Synthesis Kit (Quantabio, U.K.). The cDNA was used to assess expression of three selected microRNAs: miR-21, the main microRNA associated with pro-oncogenic function; miR-221, associated with cellular differentiation; and miR-126, which is found protective in pancreatic cancer. U6-snRNA and hsa-let-7a-5p were used as reference RNA to normalise miR expression levels. The PerfeCTa SYBR^®^ Green SuperMix (Quantabio, U.K.) was used in conjunction with MystiCq microRNA qPCR primers for miR-21 (hsa-miR-21-5p), miR-126 (hsa-miR-126-5p), and miR-221 (hsa-miR-221-5p), which were all obtained from Sigma (U.K.). The sequences for U6-snRNA primers were U6 forward, 5′-GCTTCGGCAGCACATATACTAAAAT-3′ and hsa-let-7a-5p forward 5′-CCGAGCTGAGGTAGTAGGTTGTATA-3′ reverse 5′-CGCTTCACGAATTTGCGTGTCAT-3′ for both. The conditions for thermocycling were: Denaturation at 95 °C/2 min, followed by 40 cycles at 95 °C/5 s and 60 °C/15 s, and extension at 72° C/15 s. The miR-21, miR-126, and miR-221 expression levels were normalised to that of U6 using the 2ΔΔCT method [116]. The experiments were carried out in three biological and three technical repeats.

### 4.7. Western Blotting Analysis

Total protein was extracted from treated and control-treated Panc-1 and MiaPaCa-2 cells, using RIPA + buffer (Sigma, U.K.), supplemented with 10% protease inhibitor complex (Sigma), pipetting gently at regular intervals while continuously shaking the cell preparation on ice for 2 h. Then the cell preparations were centrifuged at 16,000× *g* at 4 °C for 20 min to collect the supernatants containing the extracted proteins, which were reconstituted in 2 x Laemmli sample buffer for Western blotting. In brief, protein samples in 2 x Laemmli sample buffer containing 5% β-mercaptoethanol were boiled for 5 min at 100 °C, followed by SDS-PAGE analysis on 4–20% Mini-Protean TGX protein gels (BioRad, Watford, U.K.), and semi-dry Western blotting transfer. Ponceau S staining (Sigma) was used to assess even transfer to the nitrocellulose membranes (0.45 μm, BioRad). Blocking was carried out using 5% BSA (Sigma; in Tris buffered saline (TBS) containing 0.1% Tween20 (TBS-T)), for 1 h at room temperature (RT). Incubation with the primary antibodies was carried out overnight at 4 °C (all diluted at 1/1000 in TBS-T) as follows: anti-PAD2 (ab50257, Abcam), anti-PAD3 (ab50246), anti-PAD4 (ab50332), anti-prohibitin (ab75771), anti-moesin (ab52490), and anti-citH3 (ab5103). For EV characterisation, the EV-specific markers CD63 (ab68418; 1/1000 in TBS-T) and Flot-1 (ab41927; 1/2000 in TBS-T) were used. Washing was in TBS-T, followed by secondary antibody incubation with the corresponding HRP-conjugated anti-rabbit IgG (BioRad, U.K.) for 1 h at RT. Washing was in TBS-T and visualisation was carried out using enhanced chemiluminescence (ECL; Amersham, U.K.) together with the UVP BioDoc-ITTM System (Thermo Fisher Scientific, Hemel Hempstead, U.K.). For internal loading control and densitometry analysis of relative changes in protein expression of moesin, PHB, and citH3, the HRP-conjugated anti-β-actin antibody (ab20272, Abcam, 1/5000 in TBS-T) was used and the blots analysed with ImageJ.

### 4.8. Cancer Cell Invasion Assay

The cell invasion assay was performed according to previous methods described in detail elsewhere [17,117]. Briefly, 5 × 10^5^ cells (treated with pan-PAD inhibitor Cl-amidine and the PAD isozyme-specific inhibitors with respective PBS or DMSO control as before) were plated on Matrigel-coated transwell filters (Corning™ BioCoat™ Matrigel™ Invasion Chamber with Corning™ Matrigel Matrix; BD Biosciences, Wokingham, UK) in a chemotactic gradient of 1:10% FBS. Following an incubation time of 16 h, the number of invaded cells was determined using the crystal violet assay (Abcam, U.K.) and the MTT (3-(4,5-dimethylthiazol-2-yl)-2,5-diphenyl tetrazolium bromide) assay (Abcam, U.K.). The same number of cells was plated and incubated in parallel for 16 h, for assessment of PAD inhibitor-mediated effects on cell proliferation. The CLARIOstar plate reader (BMG Labtech, Aylesbury, UK) was used at 540–590 nm to measure absorbance and normalised according to the control. The experiments were performed in three biological and three technical repeats.

### 4.9. Statistical Analysis

GraphPad Prism version 8 (GraphPad Software, San Diego, U.S.A.) was used for statistical analysis and preparation of graphs. One-way ANOVA was used together with Tukey’s post hoc analysis. Experiments were carried out in three biological replicates for EV analysis and Western blotting, and in three biological and three technical triplicates for microRNA analysis and cell invasion assays. The generation of NTA curves was performed using the NanoSight 3.0 software (Malvern, U.K.), where the black lines in the curves represented the mean of the four repetitive 90 s readings, per individual sample (each treatment group was repeated in three biological replicates), and the red line represented the standard error (+/−). The histograms were presented as mean of data with the standard deviation (SD) indicated by the error bars. Significance was considered at *p* ≤ 0.05.

## 5. Conclusions

This is the first study to assess PAD inhibitor treatment in PDAC cells. In the current study, two pancreatic cancer cell lines (Panc-1 and MiaPaCa-2) were treated with pan-PAD inhibitor Cl-amidine alongside PAD2, PAD3, and PAD4 isozyme-specific inhibitors. PAD2 and PAD3 were found to be the dominating isoforms, and PAD inhibitor treatment affected EV signature profiles, including reducing pro-oncogenic miR-21 and miR-221, and increasing anti-oncogenic miR-126. PAD2 inhibitor, followed by PAD3 inhibitor, most effectively reduced Panc-1 cancer cell invasion and elevated moesin protein, relating to PDAC cell aggressiveness. Some reduction, but not significant, was also found in PHB levels while effects on histone H3 deimination were variable. Compared with MiaPaCa-2 cells, stronger modulatory effects for the PAD inhibitors were observed in Panc-1 cells, which importantly also showed stronger response to PAD3 inhibitor; correlating with previous observations that Panc-1 cells display neuronal/stem-like properties. Our findings report novel PAD isozyme regulatory roles in PDAC, highlighting roles for PAD isozyme-specific treatment, depending on cancer type and cancer subtypes, including in PDAC.

## Data Availability

Data is contained within the article.
